# Anti-interferon-γ autoantibodies syndrome and opportunistic infections: systematic literature review

**DOI:** 10.3389/fimmu.2025.1615091

**Published:** 2025-08-29

**Authors:** Ni Chen, Limei Hong, Hanlin Liang, Qingliang Yu, Siqiao Liang, Zhiyi He

**Affiliations:** ^1^ Department of Respiratory and Critical Care Medicine, The First Affiliated Hospital of Guangxi Medical University, Nanning, Guangxi, China; ^2^ Department of International Medical Services, The Affiliated Tumor Hospital of Guangxi Medical University, Nanning, Guangxi, China

**Keywords:** anti-interferon-γ autoantibodies syndrome, opportunistic infections, non-tuberculous mycobacteria, Talaromyces marneffei, treatment

## Abstract

**Introduction:**

Anti-interferon-γ autoantibodies (AIGAs) syndrome is a rare, adult-onset immunodeficiency characterized by increased susceptibility to various opportunistic infections. Currently, there are no standardized diagnostic or therapeutic guidelines for AIGAs syndrome, making clinical management challenging.

**Goals:**

To summarize and synthesize the published literature on the demographic characteristics, infection types, affected organs, treatments, and outcomes of AIGAs syndrome. This review aims to provide a comprehensive summary of current knowledge regarding the epidemiology, pathogenesis, clinical manifestations, diagnosis, and treatment of AIGAs syndrome.

**Methods:**

We searched PubMed, EMBASE for publications in English between January 1, 2004, and March 8, 2025 relating to AIGAs syndrome. Data from eligible studies were extracted and synthesized to summarize demographic characteristics, infection types, affected organs, treatments, and outcomes.

**Results:**

A total of 149 eligible studies, encompassing 1430 patients (664 males [46.43%], 672 females [46.99%], and 94 with unmentioned gender), were included. Patients frequently presented with two or more opportunistic infections, most commonly non-tuberculous mycobacteria (NTM, 55.69%), Talaromyces marneffei (TM, 26.98%), Salmonella spp. (12.43%), Mycobacterium tuberculosis (9.34%), and varicella-zoster virus (VZV, 9.57%). Multi-organ involvement was common, particularly affecting lymph nodes (70.24%), lungs (58.75%), bones/joints (38.89%), and skin (45.16%). Clinical outcomes included remission (45.08%), persistent (15.98%), relapse (21.62%), and death (11.68%). In addition to anti-infective therapy, corticosteroids, rituximab, and cyclophosphamide were commonly employed as immunotherapies.

**Conclusions:**

AIGAs syndrome is associated with diverse opportunistic infections, particularly NTM, TM, Salmonella, Mycobacterium tuberculosis, and VZV. Multi-organ and multi-system involvement is frequent, with a tendency toward persistent or relapsing disease and associated mortality. Corticosteroids may represent the most promising immunomodulatory therapy in the future.

## 1 Introduction

Anti-interferon-γ autoantibodies (AIGAs) syndrome is a rare clinical syndrome that predisposes individuals to infections caused by various opportunistic pathogens, such as non-tuberculous mycobacteria (NTM) species, Talaromyces marneffei (TM), Salmonella (1, 2). This syndrome is a type of adult-onset immunodeficiency, where excessive neutralizing AIGAs disrupt the binding of IFN-γ to its receptor and inhibit the JAK-STAT1 signaling pathway, thereby impairing IFN-γ-mediated immune responses (3). Unlike other immunodeficiency disorders, patients with AIGAs syndrome are selectively vulnerable to intracellular microorganisms.

The first case of AIGAs syndrome was identified in 2004 in a Filipino patient who died of severe mycobacterial infection (4). In 2012, the New England Journal of Medicine reported on adult-onset immunodeficiency in Thailand and Taiwan, revealing that neutralizing AIGAs were detected in 88% of Asian adults with multiple opportunistic infections (1). Since its initial case, numerous case and cohort studies have been documented, along with several reviews. For instance, Qiu et al. published a review study focusing on pathogen spectrum and immunotherapies from 2004 to 2021 (5). However, these studies were conducted some time ago, and recent publications have since emerged. The majority of patients are located in Asia, such as in China and Thailand, with sporadic cases reported in other regions (6, 7). In our region, we have conducted long-term cohort studies on AIGAs syndrome, with Guangxi reporting several related studies and accumulating insightful clinical management experience (8–10).

This syndrome is characterized by disseminated infections affecting multiple organs and systems. Patients often experience sequential infections with different opportunistic pathogens, leading to a protracted clinical course with tendencies for persistence and recurrence, and a heavy disease burden. Current treatments primarily involve anti-infective and immunotherapies. The immunotherapies, in particular, typically encompasses agents like rituximab and cyclophosphamide (5, 11). Recently, there has been an increase in reports on glucocorticoid therapy for AIGAs syndrome (6, 12). With a deeper understanding of the disease’s characteristics and immune features, a new review becomes valuable.

Given the absence of standardized diagnostic and therapeutic guidelines for AIGAs syndrome, this systematic review aims to summarize and synthesize published literature from 2004 to 2025 on the demographic characteristics, infection types, affected organs, treatments, and outcomes of AIGAs syndrome. This review provides a comprehensive summary of findings on the epidemiology, pathogenesis, clinical manifestations, diagnosis, and treatment of AIGAs syndrome, with the goal of aiding clinicians in better understanding and managing this condition.

## 2 Methods

### 2.1 Literature review

**Figure 1:**
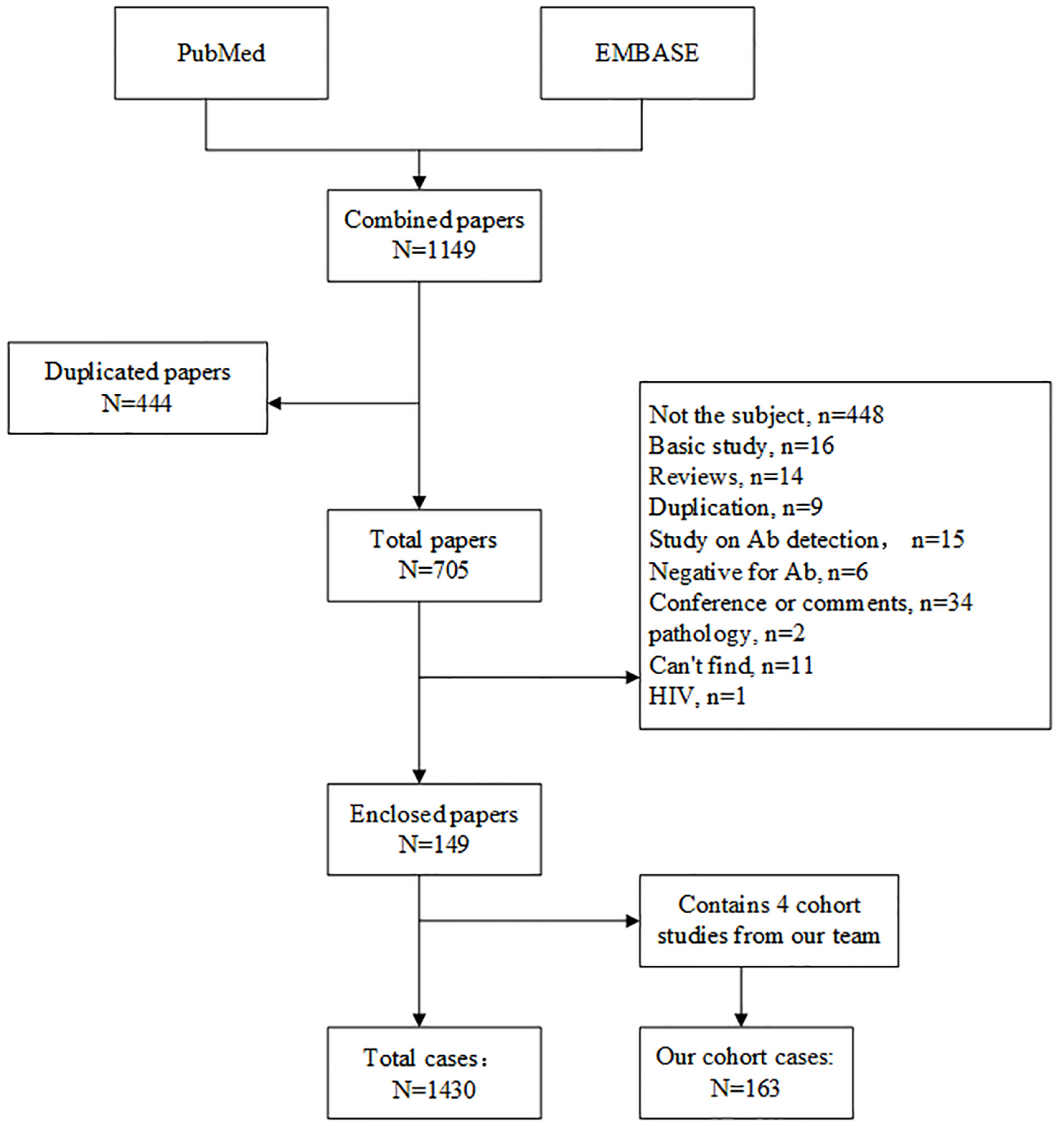
Flow chat of literature review process.

We systematically searched PubMed and EMBASE using the following terms: “anti interferon” OR “anti-interferon” OR “interferon antibody” OR “interferon autoantibody”, restricted to English-language original case reports and cohort studies on AIGAs syndrome published between January 1, 2004, and March 8, 2025. Data from eligible studies were extracted and synthesized to summarize demographic characteristics, infection types, affected organs, treatments, and outcomes. Inclusion criteria were original case reports or cohort studies with confirmed AIGAs-positive status between January 1, 2004 and March 8, 2025. Exclusion criteria were basic studies, reviews, duplicates, unrelated diseases, lacking detailed clinical records, and can’t find the full text. Literature review process was showed in **Figure 1**.

We included a retrospective cohort of 163 AIGAs-positive patients from the First Affiliated Hospital of Guangxi Medical University (located in southern China). Based on a cohort study conducted at the First Affiliated Hospital of Guangxi Medical University from January 2021 to July 2024, we collected information consistent with that from a literature review for further analysis. The Ethics Review Board of the First Affiliated Hospital of Guangxi Medical University approved the study (2024-S497-01), which was conducted in accordance with Declaration of Helsinki.

### 2.2 Definition

#### 2.2.1 Clinical outcomes definitions

The clinical course of infection was divided into the following four categories: 1) remission (complete or partial improvement of clinical symptoms after antimicrobial treatment and AIGA immunotherapy); 2) persistent infection (deterioration or no improvement of clinical symptoms after treatment); 3) relapsed infection (improvement of clinical symptoms, no pathogen detected after treatment, followed by the reappearance of pathogen-associated infectious signs and/or a positive pathogen test result); and 4) death.

Disseminated infection was defined as the involvement of two or more non-adjacent organ systems, while limited infection was defined as the involvement of only one.

Multiple microbial infections refer to infections caused by two or more opportunistic pathogens simultaneously or sequentially infecting a host.

Organ immune involvement refers to elevated levels of AIGAs titer (1:2500), accompanied by increased immune indices, such as Globulin, Immunoglobulin (Ig) G, IgE, IgG4, eosinophil and Erythrocyte Sedimentation Rate, or non-infectious clinical manifestations such as rash, immune-related ocular disorders (6).

#### 2.2.2 Statistical analysis and results

A total of 149 eligible studies published between 2004 and 2025 were systematically reviewed, including four cohorts (6, 8, 9, 12) from our institution in Guangxi, the south of China. After eliminating duplicates through integration of the largest institutional cohort (n=163), we identified 1,430 global cases of AIGA positivity. Comprehensive data extraction and statistical analyses were performed, with key findings visualized in the figures of epidemiology, geographical distribution, pathogen, outcome and immunotherapy of AIGAs. While performing the analysis there were some missing data, we assessed the overall situation by reporting the percentage or reported the number directly. The data were recorded in excel and plotted through GraphPad Prism (version 9) and Figdraw.

## 3 Epidemiology

**Figure 2:**
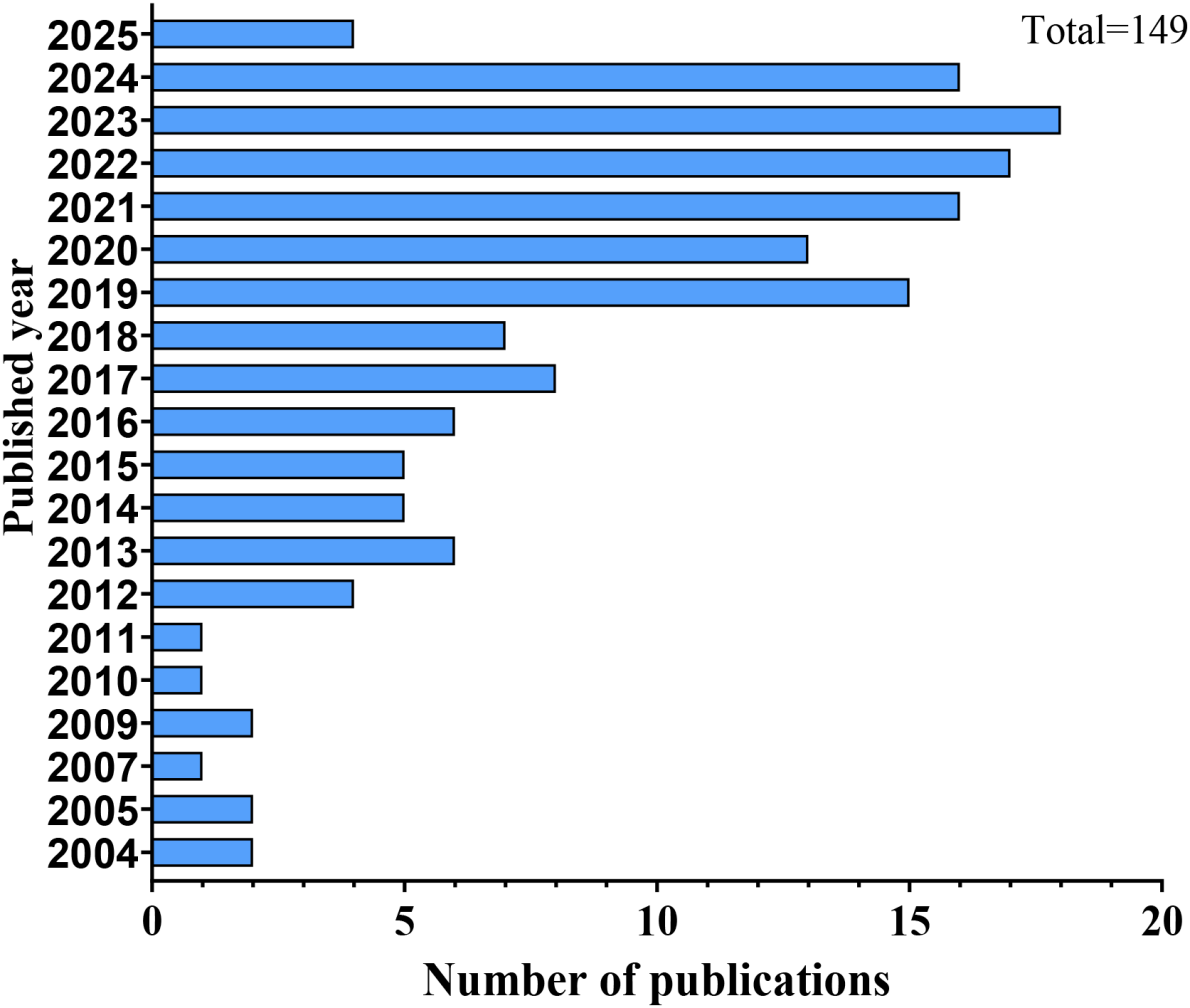
Annual publication statistics of AIGAs.

**Figure 3:**
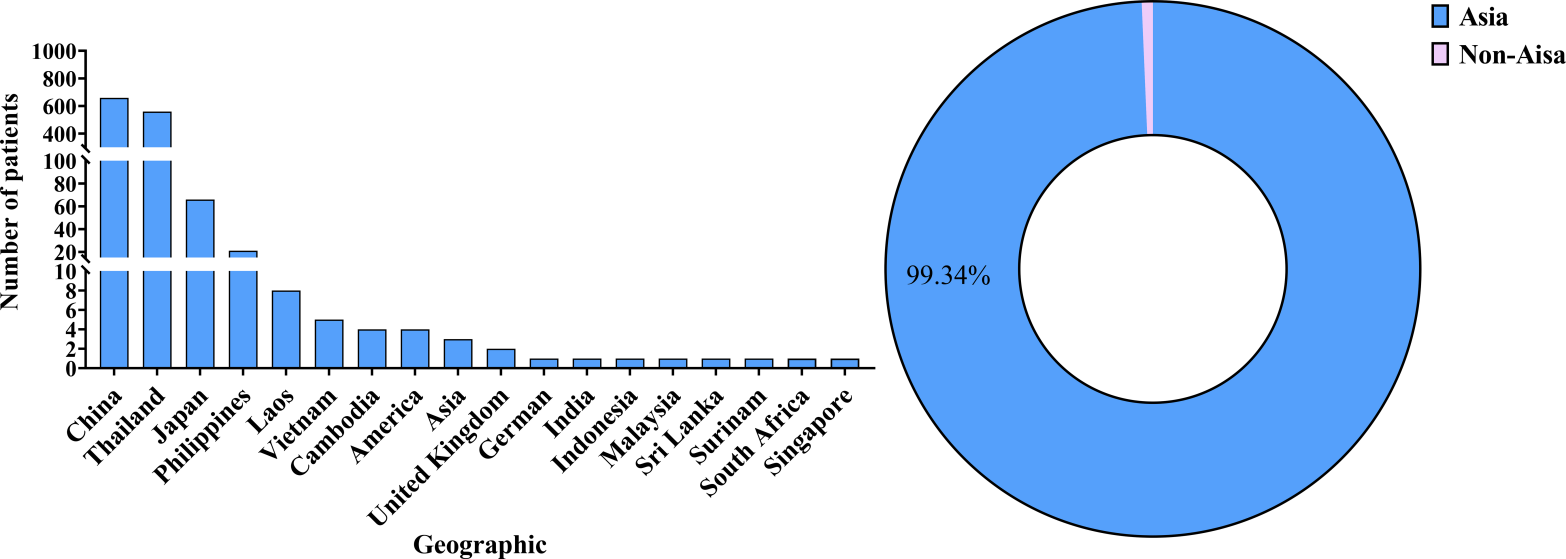
Global AIGAs case distribution.

**Figure 4:**
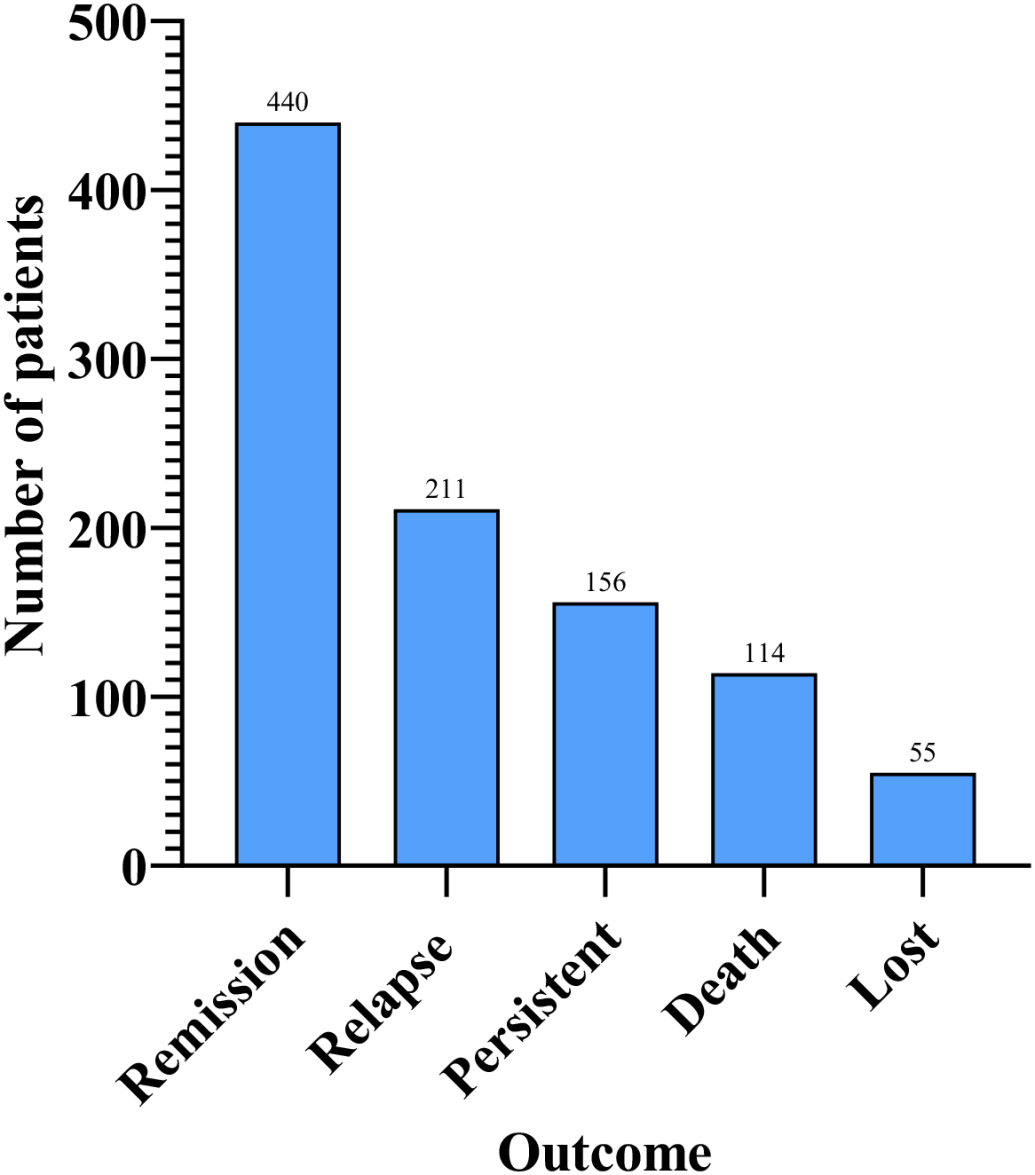
Case distribution by outcomes.

AIGAs syndrome, as a phenocopy of inborn errors of immunity (IEI) in the International Union of Immunological Societies (IUIS) classification, is associated with infections caused by mycobacteria, fungi, Salmonella, and VZV due to excessive autoantibodies against IFN-γ (13). Since the first case of severe mycobacterial infection in 2004 (4), 1430 cases of AIGAs syndrome have been documented globally. Previous studies were predominantly case reports. However, in recent years, the number of publications has increased, with a notable rise in cohort studies. (**Figure 2**) The prevalence of the disease is predominantly observed in regions of southern China, such as Guangxi and Taiwan, as well as in other Aisa countries including Thailand and Japan (14–18). (**Figure 3**) Scattered cases have also been documented in non-Asian regions, such as the United Kingdom, the United States, and Germany (19–21). (**Figure 3**) Browne et al. found AIGAs were detected in 88% of Asian adults with multiple opportunistic infections (1). The prevalence of AIGAs is notably high (94.8%) among patients with HIV-negative disseminated TM infection (15). However, no large-scale epidemiological investigation has accurately determined its morbidity and mortality rates, and the incidence of this disease is significantly underestimated due to insufficient clinical awareness. Due to immunodeficiency, the disease is often combined with recurrent and persistent opportunistic infections, leading to a poor prognosis. Zhang et al. reported that the recurrence rate of AIGAs syndrome was as high as 56.0% (22). Wongkularb et al. reported that in northern Thailand, patients positive for AIGAs had a relatively high mortality rate, with 32% of patients dying at a median time of 25 months after diagnosis (23). According to the literature review, the outcomes data showed the following proportions: remission in 45.08% of patients, persistence in 15.98%, relapse in 21.62%, and death in 11.68% of patients, respectively. (**Figure 4**) Late diagnosis, persistent and recurrent severe infections, and the lack of standardized treatment may result in a poor prognosis for patients with AIGAs. Wu et al. indicated a misdiagnosis rate of approximately 33%, because part of cases were misidentified as tuberculosis, metastasis, connective tissue diseases, or lymphoma (24, 25). The mean age at diagnosis is approximately 53 years, based on the literature review. A small number of cases of occurred in young age (26). There was no significant gender difference (6). AIGAs syndrome has a long-term course, persisting for more than 28 months (22). Future efforts should focus on enhancing and universalizing disease diagnostic techniques, as well as implementing prospective epidemiological surveillance, to promote awareness and management of AIGAs syndrome and alleviate its disease burden.

## 4 Pathogenesis

The pathogenesis of AIGAs syndrome remains poorly understood, hypothesized to arise in genetically predisposed hosts following exposure to various environmental triggers. AIGAs belong to the IgG class and predominantly consist of IgG1, IgG3 and IgG4 subcalsses (27). AIGAs can be detected in the serum of healthy individuals, with low titers lacking neutralizing capacity. Conversely, AIGAs titers are elevated and possess neutralizing capabilities in AIGAs syndrome, which can sustain *in vitro*. The current studies have not elucidated how antibodies become a trigger for pathogenicity. Molecular mimicry stands as one of the theories positing that AIGAs emerge in response to the immune system reacting to foreign antigens sharing similar sequences or structures with endogenous antigens. Lin et al. demonstrated that the epitope P121–131 (SPAAKTGKRKR) at the C terminus of IFN-γ displayed a high degree of sequence homology to the Aspergillus Noc2 protein, which is also recognized by AIGAs, the treatment with epitope-erased variant of IFN-γ (EE-IFN-γ) might be effective in patients with AIGAs (28). Genetic factors are currently confirmed to be closely related to the pathogenicity of AIGAs. Most of southeast Asia AIGAs patients have been found to possess HLA-DR*15:02/16:02 or HLA-DQ*05:01/05:02 (15, 29). Nevertheless, Peel et al. thought that neutralizing AIGAs occur sporadically, with low penetrance observed in individuals carrying the HLA-DRB1*15:02 or 16:02 alleles, indicating a potential association with rare germline or somatic variants (27). The disparity in observed outcomes may stem from variations in the populations under cohort observation. Over all, the pathogenicity of AIGAs may be the result of environment and gene, the exact mechanism is not clear.

**Figure 5:**
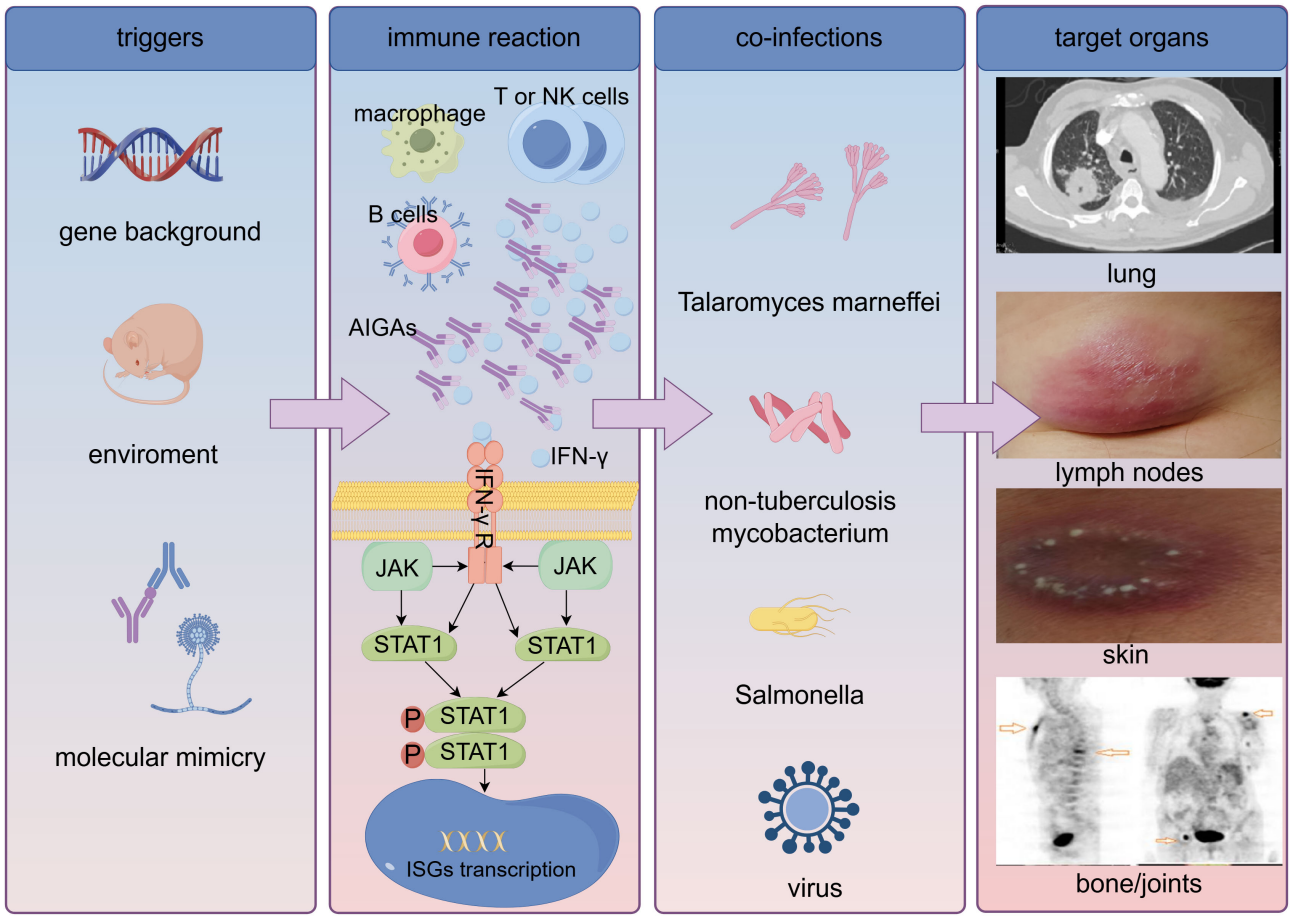
Pathogenesis of anti-interferon-γ autoantibodies syndrome. AIGAs, anti-interferon-γ autoantibodies; IFN-γ, interferon-γ; IFN-γ R, IFN-γ receptor; ISGs, IFN-stimulated genes.

IFN-γ, secreted by various immune cells such as activated T cells, natural killer (NK) cells, and macrophages, plays a crucial role in normal immune surveillance, exhibiting immunomodulatory, antimicrobial, and anticancer properties. Deficiencies in peripheral tolerance mechanisms of B cells, combined with somatic hypermutation resulting in the emergence of autoreactive B cells exhibiting increasingly strong binding to self-antigens, could potentially lead to an overproduction of AIGAs (2). High-titer AIGAs inhibit the binding of IFN-γ to its receptor, consequently blocking JAK-STAT1 pathway and downstream biological responses, including ISGs (IFN-stimulated genes) transcription, M1 macrophage polarization, IFN-γ-regulated inflammation, chemokine production, and cytokine production (3, 30, 31) (**Figure 5**).

Yuan et al. reported a downregulation of the C-X-C motif chemokine ligand 9 (CXCL9) gene in patients with AIGAs, and CXCL9 served as a dependable biomarker to distinguish lymphadenopathy associated with AIGAs, which mimicked lymphoma (32). Chemokines may influence the pathogenesis of AIGAs syndrome by inducing the migration and directional proliferation of immune cells through various mechanisms, the research in this area remains limited. Additionally, AIGAs have been shown to inhibit the upregulation of HLA class II expression on peripheral blood mononuclear cells, thereby affecting antigen presentation and immune regulation (19). The findings of Shih et al. suggested that pathogenic AIGAs function by blocking IFN-γ signaling and eliminating IFN-γ-responsive cells via Fc-mediated responses (33). Moreover, the immune systems of patients with AIGAs may exhibit T cell depletion and an adaptive increase in NK cells, potentially associated with alterations in the Th1/Th2 balance (34, 35). However, the specific mechanisms require further investigation. In summary, neutralizing AIGAs impede the effects of IFN-γ, resulting in the disruption of downstream signaling pathways and impacting the immune response to intracellular pathogens.

## 5 Clinical manifestations

**Figure 6:**
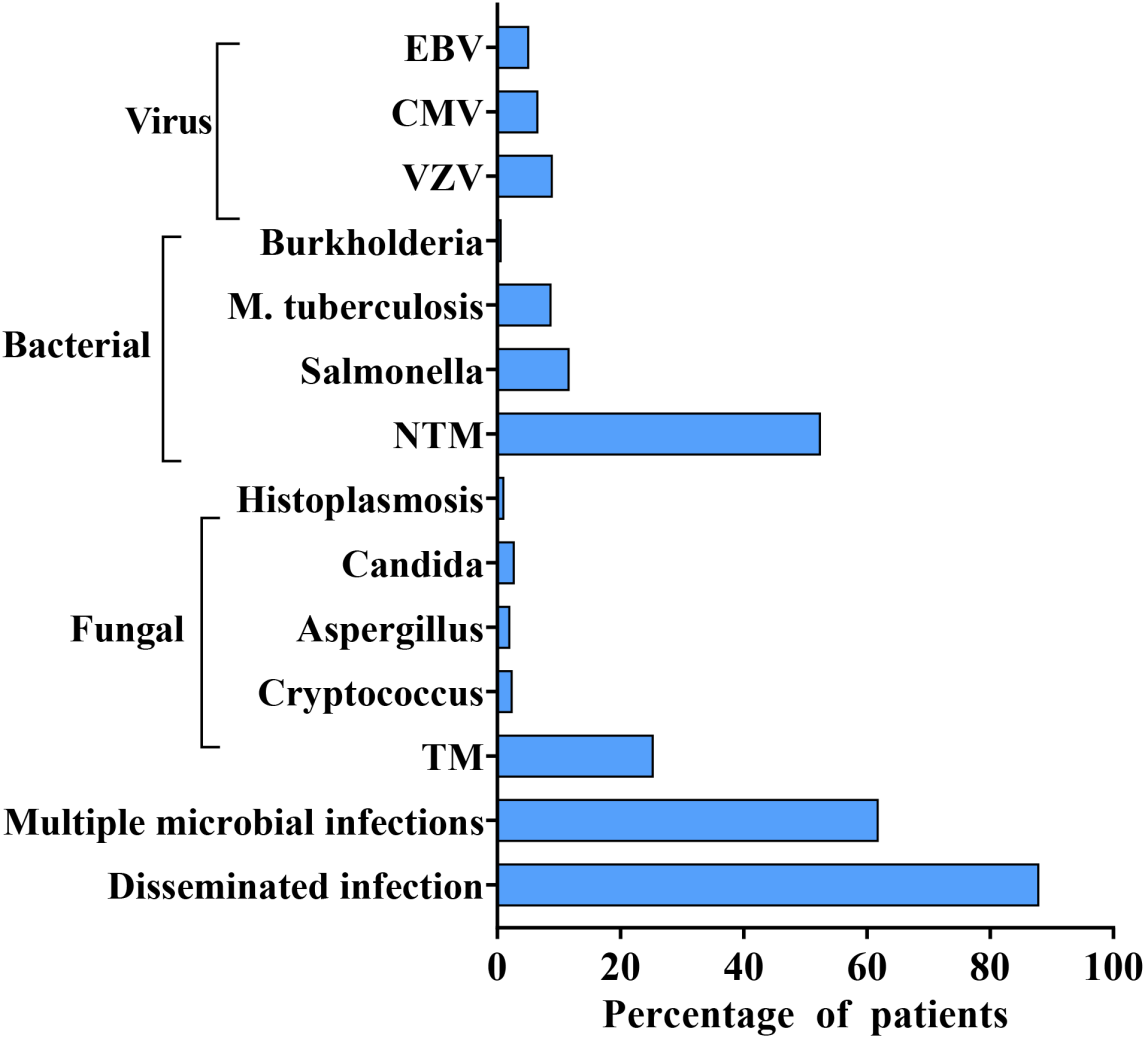
Percentage of primary pathogens in AIGAs patients.

**Figure 7:**
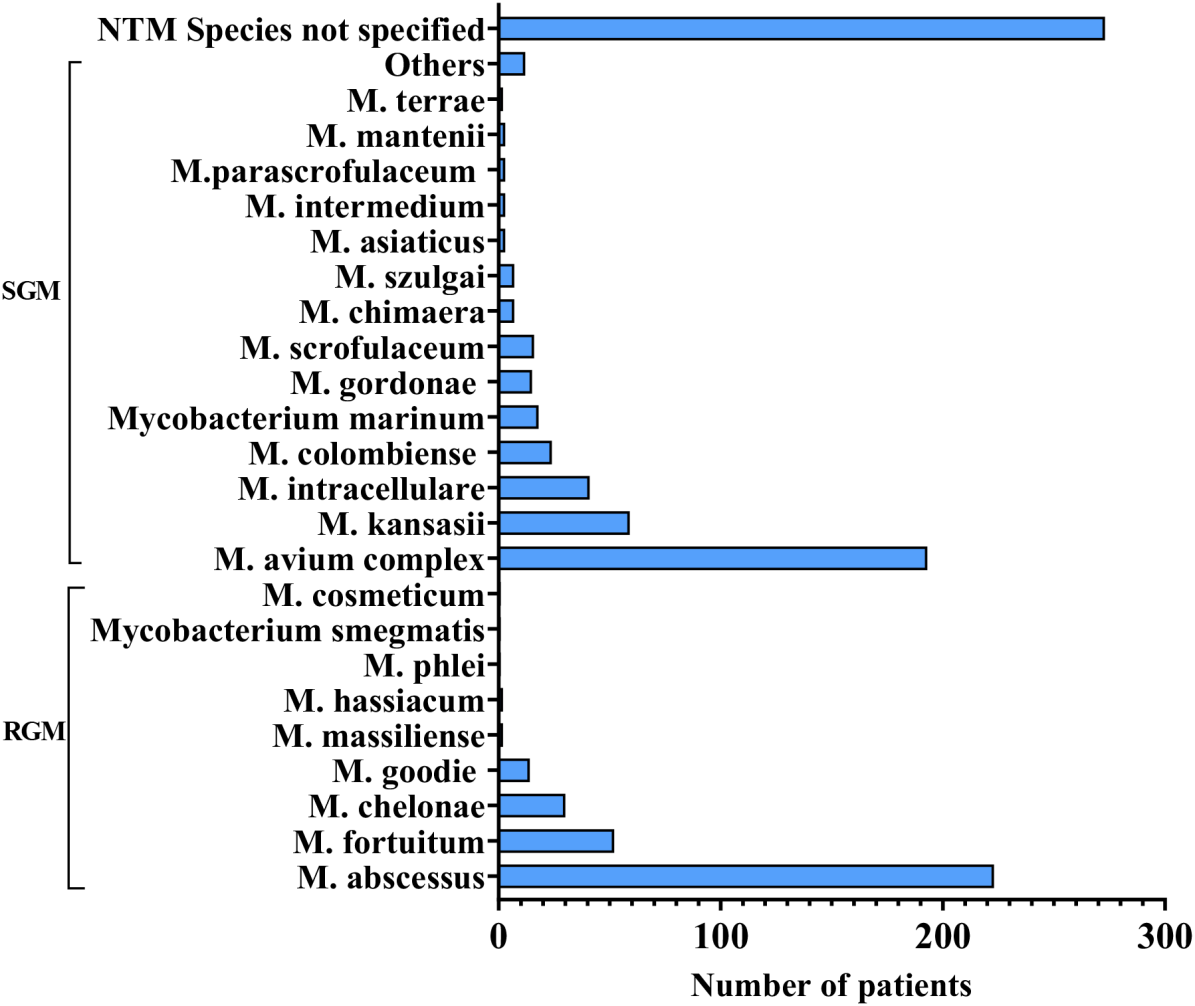
NTM species stratification in AIGAs patients.

Disseminated opportunistic infections frequently serve as the initial diagnostic clue for AIGAs syndrome. The disease is commonly associated with two or more pathogens, including NTM (55.69%), TM (26.98%), Salmonella spp. (12.43%), Mycobacterium tuberculosis (9.34%), and varicella-zoster virus (VZV) (9.57%), based on the literature review. (**Figure 6**) NTM and TM are the most common opportunistic pathogens. Chi et al. reported that the positive rate of AIGAs reached up to 97.8% in disseminated NTM infections. Common species of NTM are Mycobacterium abscessus and Mycobacterium avium complex (MAC) (9, 25). (**Figure 7**) Lung and bone involvement are common organs involved in patients with slow growth NTM such as MAC, whereas lymph nodes and skin lesions are more common in patients with rapidly growing mycobacteria (RGM), such as Mycobacterium abscessus (16, 36). Chi et al. reported that AIGAs may play a critical role in the pathogenesis of disseminated NTM infections and reactivation of latent VZV infection (17). In summary, AIGAs are highly associated with opportunistic infections in non-HIV patients and may contribute to the development of severe, complicated infections.

**Figure 8:**
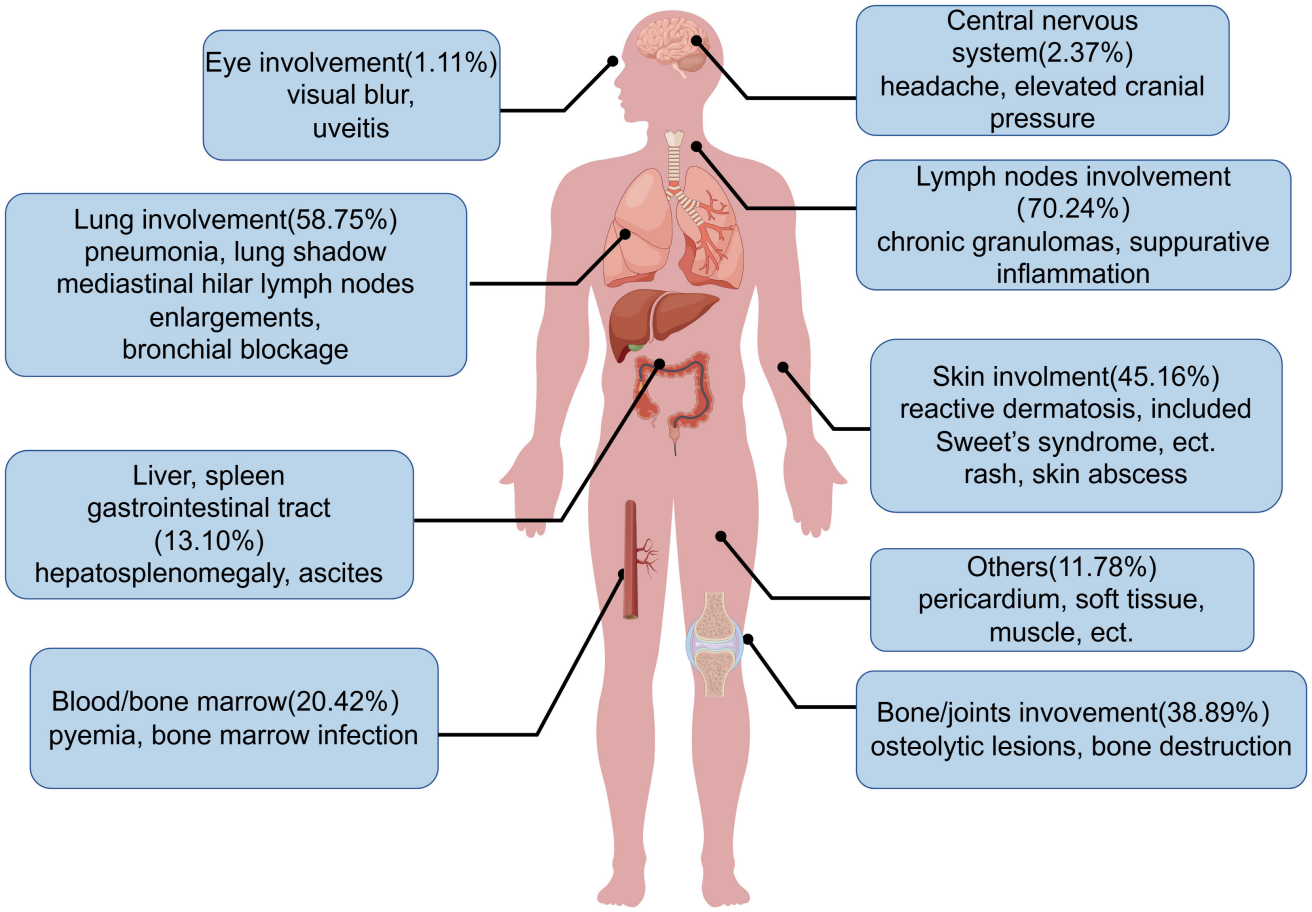
Affected organs in anti-interferon-γ autoantibodies syndrome. Shown are the affected organs with the frequency, and common clinical manifestations in anti-interferon-γ autoantibodies syndrome.

The initial clinical symptoms or signs of AIGAs-positive patients mainly stem from opportunistic infections, with disseminated infections typically leading to systemic lymphadenitis and skin lesions. Common clinical manifestations include unexplained fever, emaciation, cough and bone pain. Overall, AIGAs syndrome can affect various organs, including lymph nodes (70.24%), lungs (58.75%), bone/joints (38.89%), skin (45.16%), based on the literature review (14, 15, 25). (**Figure 8**) Additionally, some cases involved bone marrow/blood, liver and spleen, throat, eyes, central nervous system (21, 37–42). In a small number of patients, bronchial involvement may occur, with the rupture of mediastinal lymph nodes potentially leading to broncho esophageal fistula, or lymph node enlargements or granulomas obstructing the bronchi (43, 44). Proliferative lesions are typically caused by TM or slow-growing NTM such as Mycobacterium avium, Mycobacterium kansasii. A study reported a 6% incidence of bronchial lesions in 111 NTM patients with AIGAs (45). Besides, skin involvement is common in the AIGAs syndrome, which can be categorized into reactive and infectious skin lesions based on the etiology. A study reported that 39% of AIGAs-positive patients experienced reactive neutrophilic dermatosis during the disease course (46). Results from Pattanaprichakul et al. suggested that reactive dermatosis as the predominant skin manifestation in AIGAs syndrome patients, occurring in 66.1% of cases, followed by disseminated (18.3%) and localized skin infections (15.6%) (36). Reactive dermatosis, primarily observed on the upper body and linked with disseminated infection, included Sweet’s syndrome, erythema nodosum, lobular panniculitis, and various generalized pustular eruptions (47). According to the literature review, Sweet’s syndrome occurred in 15.12% of patients with AIGAs. Such reactions usually show positive responses to immunomodulating agents, like glucocorticoid. In contrast, non-reactive dermatosis refers to skin infections, often resulting in chronic granulomas or suppurative inflammatory lesions, which typically improve with antimicrobial therapy.

Lymph nodes involvement is prevalent among patients with AIGAs, and the enlargement of multiple lymph nodes presents challenges in distinguishing between lymphoma and IgG4-related diseases based on clinical manifestations and pathology. Histopathological analysis of lymph node biopsies typically reveals chronic granulomas and suppurative inflammation. One study identified three histomorphologic patterns in lymph node biopsy specimens from patients with disseminated non-tuberculous mycobacterial infection: well-formed granulomas (46%), suppurative inflammation or loose histiocytic aggregates (31%), and lymphoproliferative disease (LPD, 23%) (48). Another study by Thingujam et al. demonstrated that poorly defined granulomas and neutrophil clusters adjacent to histiocyte aggregates strongly suggested AIGAs (49). AIGAs can co-occur with both lymphoma and IgG4-related diseases (50, 51), although such occurrences are relatively rare. Chi et al. showed that malignancy in four patients concurrently diagnosed with AIGAs, all originating from the T cell/macrophage lineage (52). AIGAs syndrome can often be mistaken for other diseases due to similarities in clinical manifestations and pathology, conducting expedited pathogen identification tests alongside histopathological examinations facilitates timely and accurate diagnosis, thereby enhancing prognosis.

### 5.1 Diagnosis

**Figure 9:**
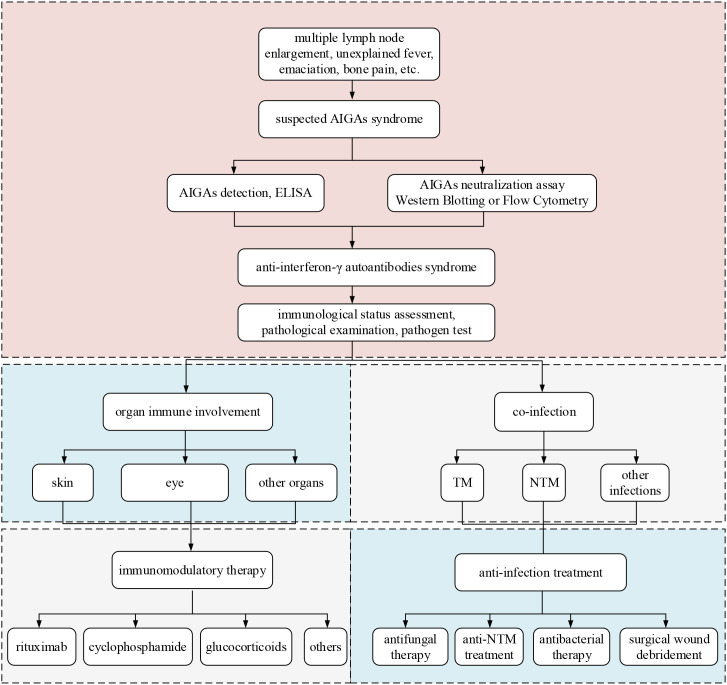
Diagnostic process and treatment of anti-interferon-γ autoantibodies syndrome.

At present, there are no established clinical guidelines domestically or internationally for diagnosing AIGAs syndrome. Diagnosis is primarily reliant on the patient’s clinical presentation and detection of AIGAs. (**Figure 9**) In cases patients exhibit more than two types of complex opportunistic infections, particularly intracellular bacterial infections like NTM and TM, involving multiple organs and systems such as lymph nodes, lungs, bones, and skin, elevated serum levels of AIGAs and their neutralizing capacity indicate the presence of AIGAs syndrome. Clinical indicators such as elevated inflammatory markers, and high serum IgG and globulin levels strongly suggest the presence of AIGAs syndrome (6). It is crucial to differentiate from other diseases, including neoplastic conditions such as lymphoma, as well as non-neoplastic diseases such as Mendelian susceptibility to mycobacterial diseases (MSMD), Granulomatous-Lymphocytic Interstitial Lung Disease (GLILD), sarcoidosis, IgG4-related diseases (53–57). (**Supplementary Table S1**) Pathological and etiological examinations are essential for achieving a definitive diagnosis. Notably, MSMD must be differentiated from AIGAs syndrome, as both can present with lymphadenopathy, fever, and sepsis. However, MSMD typically manifests at an earlier age (onset age: 10.41 ± 0.42 years) and is commonly associated with monogenic mutations, such as IL12RB1 or IFNGR1 (53). Genetic testing aids in differential diagnosis.

Patients suspected of having AIGAs syndrome should be evaluated based on clinical manifestations, such as multiple lymph node enlargement, unexplained fever, emaciation, bone pain, as well as findings of opportunistic pathogens, particularly infections with TM or NTM. These patients should undergo serum screening for AIGAs. Elevated titers of AIGAs, confirmed by enzyme-linked immunosorbent assay (ELISA), and their neutralizing activity, confirmed by western blotting or flow cytometry, are diagnostic criteria. Positive findings for AIGAs warrant an assessment of the patient’s immunological status, including levels of immunoglobulins and lymphocyte subsets, as well as pathological examination and pathogen testing. Organ immune involvement often affects the skin, eye and may extend to systemic immune responses and involvement of other organs (6, 12, 36, 39, 58). Immunotherapy include treatments with rituximab, cyclophosphamide, glucocorticoids, and other medications. Co-infections are frequently caused by TM and NTM. Anti-infection treatment must be tailored to the specific pathogen, encompassing antifungal therapy, anti-NTM therapy, and antibacterial therapy. In cases with abscess formation or poor drug response, surgical intervention may be considered.

The detection of AIGAs involves (1) determining the titer or concentration of AIGAs and (2) assessing their neutralizing activity. Some studies have suggested that QuantiFERON-TB Gold In-Tube (QFT-GIT) IFN-γ release tests may indicate low IFN-γ expression or undetermined results suggesting possible AIGAs positivity, which can be used as a preliminary detection method (59). It remains unclear whether T-SPOT can similarly serve a predictive role. At present, it is common to use ELISA indirect method, sandwich, or inhibition method test, which can be used to detect antibody qualitatively or by OD value (15, 17, 60). The evaluation of pSTAT1, the downstream element on the Janus-activated kinase (JAK)-STAT pathway, is a commonly important method to confirm the neutralizing activity of AIGAs, and is usually performed by Flow Cytometry or Western Blotting (1, 7, 17, 61). However, all these techniques are time-intensive or necessitate costly equipment, AIGAs detection is mainly concentrated in scientific research institutes or testing companies, which may indirectly improve the rate of misdiagnosis in basic hospitals. Improving and popularizing the detection techniques for AIGAs is currently needed.

## 6 Treatment

The AIGAs syndrome is accompanied by variable opportunistic infections among patients. Currently, the main therapeutic methods consist of anti-infection therapy and immunomodulatory therapy. The overall goal is prompt reduction of antibody titers, control of excessive immune reaction, and management of infections to prevent relapses. Immunomodulatory may sustain low antibody titers for an extended period, leading to disease remission.

### 6.1 Anti-infection treatment

The AIGAs syndrome initially manifests as an infection, and anti-infection measures are crucial in disease management. Early and precise diagnosis, facilitated by various pathological detection methods (such as culture, next-generation sequencing, etc.), is essential for effective treatment strategies and improving prognosis (43, 62). Treatment of TM with AIGAs typically involves intravenous administration of amphotericin B, followed by oral itraconazole or voriconazole (63). For patients who are unable to tolerate amphotericin B due to hepatic or renal function impairment (64), sequential oral treatment following intravenous voriconazole is recommended. Anti-NTM treatment is selected based on strain identification and drug susceptibility results (65, 66). Common oral drugs for anti-NTM treatment include azithromycin, clarithromycin, ethambutol, rifampin, moxifloxacin, and linezolid, while intravenous options include imipenem, tigecycline, and amikacin. Omadacycline and phage therapy are novel treatments for drug-resistant mycobacterial disease (67, 68). For patients exhibiting extensive lesions, abscess formation, and inadequate response to medication, surgical wound debridement is advised (69, 70). Patients with AIGAs syndrome typically require longer treatment courses compared to those with normal immune function, lasting at least one and a half to two years. These patients are prone to recurrent infections, including those caused by new or drug-resistant pathogens. Specific antimicrobial regimens, antimicrobial durations, and criteria for discontinuation of drugs in AIGAs syndrome need to be further clarified.

### 6.2 Immunomodulatory therapy

**Figure 10:**
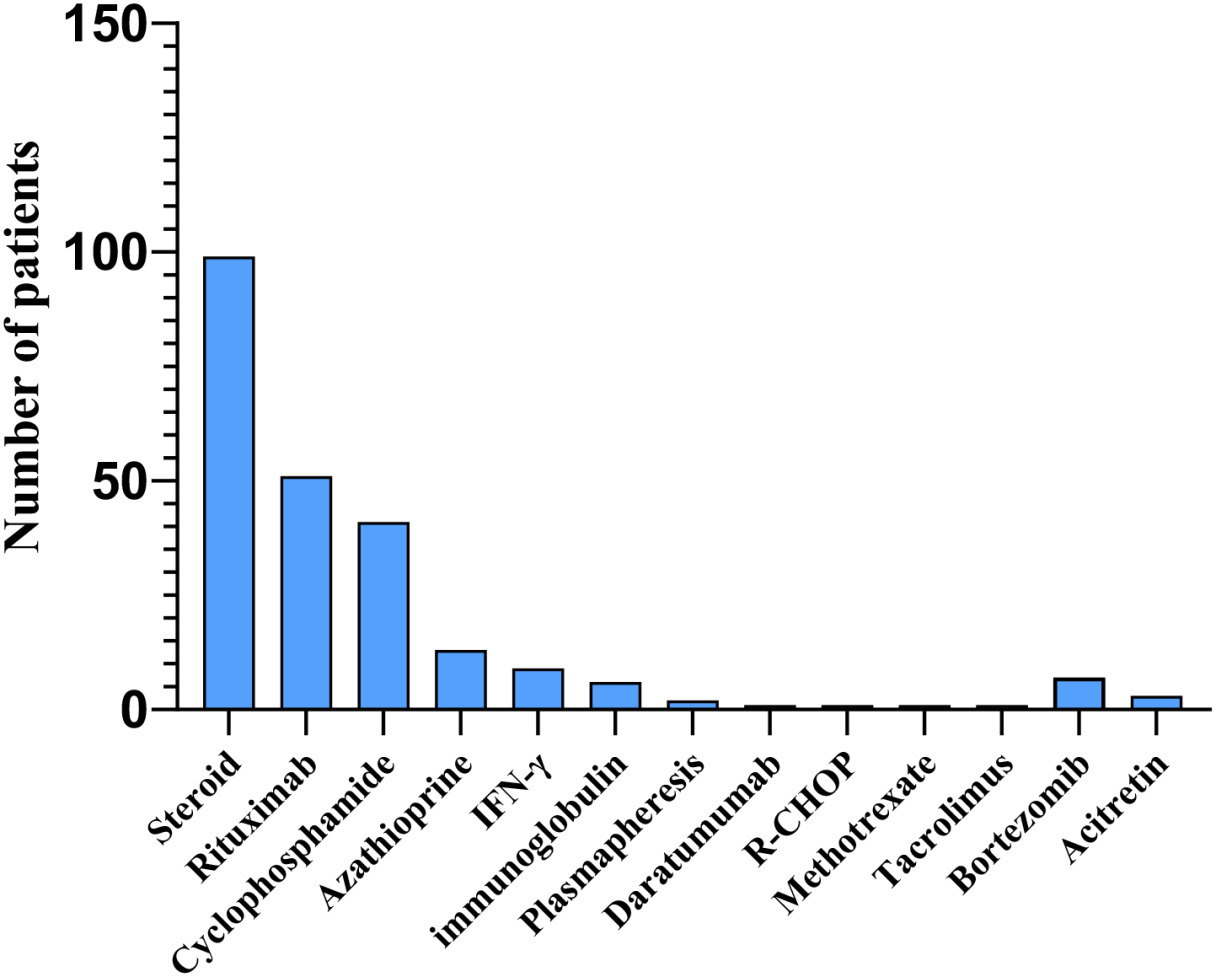
Distribution of patients receiving different immunotherapies in AIGAs.

Despite prolonged and rigorous antimicrobial therapy, many patients with AIGAs suffer from frequent disease relapses. Immunomodulatory therapy is employed as an adjuvant treatment for anti-infection therapy, demonstrating therapeutic efficacy. This therapy typically includes glucocorticoids, rituximab, cyclophosphamide, azathioprine, bortezomib, with a small proportion of studies reporting the use of immunoglobulin, daratumumab, plasma exchange, and interferon treatment (71–75). (**Figure 10**) According to the literature review, a total of 177 patients received immunotherapy, among whom 36 (20.57%) were treated with at least two different immunotherapeutic agents. B cell depletion therapy with rituximab offered a targeted therapeutic approach for patients with high-titer AIGAs who exhibit progressive refractory nontuberculous mycobacterial disease despite anti-infection treatment (61, 76). This therapy has been shown to reduce AIGAs levels and restore IFN-γ signaling, as evidenced by IFN-γ-induced STAT1 phosphorylation (77). However, the timing and regimen of rituximab administration remain unknown and require further investigation. Studies have demonstrated that intravenous cyclophosphamide effectively reduced AIGAs titers in patients with high AIGAs levels and refractory infections, although some patients experienced relapses (78, 79). The report of Laisuan looking at the prospective trial of biosimilar anti-CD20 therapy is based on nonstandard dosing of the drug compared with dose escalation of cyclophosphamide (11). Laisuan et al. found that cyclophosphamide treatment exhibited superior therapeutic efficacy compared to rituximab, demonstrating a tendency towards faster symptom relief, prolonged remission periods, and a decreased incidence of relapse infections (11). Glucocorticoids are commonly used immunomodulatory agents that exert anti-inflammatory, immunoregulatory, and anti-allergic effects. Glucocorticoids therapy is mainly utilized for patients with reactive skin lesions (80). Several cohort studies have revealed that a high titer of AIGAs, elevated levels of globulin, immunoglobulin G, IgE, IgG4, eosinophils, and erythrocyte sedimentation rate, as well as non-infectious clinical manifestations such as rashes and immune-related ocular disorders, are indicative of immune damage, glucocorticoids have demonstrated therapeutic efficacy in managing these conditions (6, 12). Recently, a study indicated that treatment with bortezomib followed by cyclophosphamide did not lead to a significant decrease in AIGAs titer levels, and 10 opportunistic infections were observed during 24–72 weeks of bortezomib treatment (7). In future, large prospective cohort studies or randomized controlled trials are still necessary to investigate the timing, duration, and efficacy of immunomodulatory therapy for AIGAs syndrome.

### 6.3 Disease monitoring and efficacy indicators

It is suggested to monitor inflammatory indicators, AIGAs and immune indicators including IgG, IgE, total globulin, and globulin levels in patients with AIGAs syndrome (6). The study by Loh et al. proposed the necessity of detecting antibodies in NTM infection with more than two opportunistic infections or reactive skin diseases (81). Serum antibody titers fluctuate throughout the course of the disease and are not stable during treatment, which correlates with disease progression (18). AIGAs titers serve as important indicators affecting the efficacy and outcome of patients. AIGAs positivity and disease activity were associated with multiple opportunistic and disseminated infections, high white blood cell counts, elevated C-reactive protein levels, increased erythrocyte sedimentation rates, as well as neutrophils, low hemoglobin levels, elevated serum IgG and globulin levels, and decreased CD4+ T cell counts (14, 82). AIGAs syndrome presents with a prolonged course characterized by persistent and recurrent infections. Monitoring AIGAs titers, inflammatory indicators, and immune indicators are crucial for assessing the patients’ condition and guiding treatment.

## 7 Discussion

We performed a systematic review of AIGAs syndrome, and provided a comprehensive summary of findings on the epidemiology, pathogenesis, clinical manifestations, as well as diagnosis and treatment for AIGAs syndrome. From 2004 to 2025, our review included 1,430 patients from 149 studies. These patients originated from 17 countries globally, predominantly from Asian regions such as Guangxi in China, Thailand, and Japan. Guangxi has emerged as the region with the highest number of reported cases and the largest cohort for AIGAs syndrome. This may be attributed to the tropical and subtropical climate in southern China, which fosters the growth of numerous fungi and other microorganisms. Studies have indicated that both soil exposure and the rainy season may contribute to the increased dissemination of TM conidia (83, 84).

Additionally, certain local dietary habits, such as the consumption of or contact with bamboo rats, may increase the risk of exposure to and infection with TM, a pathogen hosted by bamboo rats (85). Consequently, a higher number of TM infection and AIGAs syndrome patients have been identified in Guangxi, enhancing the diagnostic rate for this syndrome. Genetic factors also play a role, as most Southeast Asian AIGAs patients have been found to possess HLA-DR15:02/16:02 or HLA-DQ05:01/05:02, as literatures reported (15, 17). Thus, AIGAs syndrome is believed that caused by a combination of genetic and environmental factors.

AIGAs syndrome exhibits clinical features resembling those observed in MSMD, such as heightened susceptibility to NTM infections and other related manifestations. However, the pathogenic mechanisms differ. MSMD is caused by genetic mutations in the IFN-γ signaling pathway, which impair immune responses to mycobacteria and typically manifest in childhood (53). In contrast, AIGAs syndrome is an adult-onset immunodeficiency syndrome where increased levels of neutralizing AIGAs block the IFN-γ signaling pathway, preventing it from functioning properly. Regarding the pathogenic mechanisms of neutralizing antibodies, Shih et al. demonstrated that AIGAs can bind to Fcγ receptors via their Fc region, triggering antibody-dependent cellular cytotoxicity (ADCC) that targets and eliminates IFN-γ-responsive cells (33). Their experiments showed that monoclonal antibodies targeting site III of IFN-γ efficiently induce ADCC, leading to a significant reduction in IFN-γ signaling. Moreover, AIGAs can form immune complexes that block IFN-γ receptor binding and inhibit downstream signaling activation. These findings highlight the critical role of Fc-mediated mechanisms in the pathogenesis of AIGAs. Further research is needed to better understand the mechanisms underlying the production and pathogenicity of neutralizing antibodies.

The clinical manifestations of AIGAs syndrome are primarily associated with opportunistic infections, often involving two or more opportunistic pathogens and disseminated infections. Common opportunistic pathogens include NTM, TM, Salmonella, Mycobacterium tuberculosis, and VZV. AIGAs syndrome can affect a range of organs, with the lymph nodes, lungs, bones/joints, and skin being the most commonly affected sites. These findings align with those reported in the literature. Additionally, we analyzed the clinical outcomes of AIGAs syndrome patients reported in the literature. Remission occurred in 45.08% of patients, persistent infections in 15.98%, relapses in 21.62%, and mortality in 11.68%. Variations in outcomes may be due to differences in follow-up duration and regional factors. For example, Liang et al. and Chen et al. reported mortality rates of 7.41% and 11.04%, respectively, in Guangxi, China, over three years of follow-up (6, 12). In contrast, Wongkularb et al. observed a higher mortality rate of 32% in northern Thailand, with a median survival of 25 months (23). Currently, no large-scale epidemiological studies have assessed survival rates, but available data suggest a poor prognosis for AIGAs patients.

Immunological features of AIGAs syndrome include elevated AIGAs, IgG, IgG4, IgE, and erythrocyte sedimentation rate. Liang et al. classified the clinical course of AIGAs syndrome into two phases: an infective phase and a stable phase. The stable phase is further subdivided into three types: Type I, characterized by low AIGAs titers without immune damage; Type II, with high AIGAs titers but no immune damage; and Type III, featuring high AIGAs titers accompanied by immune-mediated damage (6). Glucocorticoids have demonstrated favorable therapeutic efficacy, particularly in patients with high antibody titers and associated immune damage. Regarding immune cell alterations, some studies suggest that the immune systems of patients with AIGAs may exhibit T cell depletion and an adaptive increase in NK cells, potentially associated with alterations in the Th1/Th2 balance (34, 35). The persistent decrease in CD4^+^ T cell counts may indicate worsening immune exhaustion, NK cell subsets can provide a basis for immune regulatory therapy. The study by Liang et al. also indicated that CD4^+^ T cell counts were lower in infected patients who were negative for AIGAs, while NK cell counts were slightly higher (6). These findings may contrast with existing literature and warrant further discussion. Monitoring immunological manifestations at different phases and proposing threshold values for disease progression or changes will be the direction of our future research.

Currently, treatment of AIGAs syndrome primarily involves antimicrobial therapy combined with immunotherapy. Previous literature has reported numerous cases using rituximab and cyclophosphamide, which have demonstrated efficacy in reducing antibody levels and improving symptoms in some patients. However, these therapies have limitations, including potential further immune suppression and disease relapse upon discontinuation. This review highlights glucocorticoids as the most commonly employed immunotherapy, particularly effective in patients with reactive skin manifestations and immune-mediated damage, such as Sweet’s syndrome. Glucocorticoids may represent the most promising immunotherapeutic approach for AIGAs syndrome in the future.

## Limitations

In summary, this is a comprehensive systematic review of AIGAs syndrome, covering epidemiology, pathogenesis, clinical manifestations, diagnosis, and treatment. It holds significant importance for clinicians and researchers in diagnosing and treating this condition. However, there are some limitations. First, the inclusion of a small number of duplicated cases are unavoidable. Second, the discussion of pathogenesis is not in-depth enough. Lastly, the efficacy of immunotherapy and AIGAs titer change cannot be fully assessed. In the future, it is essential to popularize AIGAs detection technologies, conduct large-scale epidemiological surveys, delve deeper into pathogenesis, and further explore new immunotherapies and related research on immunotherapy.

## Conclusions

AIGAs syndrome is a type of immune deficiency characterized by high titers of AIGAs with neutralizing ability. This syndrome leads to disseminated opportunistic infections involving multiple organs or systems, such as lymph nodes, lungs, bones, skin, and blood. Chronic recurrent infections compounded by immunocompromised states often result in a poor prognosis. Early diagnosis, along with monitoring antibody titers and inflammatory and immune indices, can improve prognosis. The typical treatment approaches involve anti-infection therapy and immunomodulatory therapy. However, further researches are needed to improve diagnostic tools and determine the optimal timing, duration, and effectiveness of immunomodulatory therapy for managing AIGAs syndrome.

## Data Availability

The original contributions presented in the study are included in the article/**Supplementary Material**. Further inquiries can be directed to the corresponding authors.
