# Supplementary material for: Anti-interferon-γ autoantibodies syndrome and opportunistic infections: systematic literature review
Source: Front Immunol. 2025 Aug 29;16:1615091. doi: 10.3389/fimmu.2025.1615091 (PMC12426263; doi:10.3389/fimmu.2025.1615091)
Supplement: Supplementary file 1 [file Table1.docx]

Supplementary Table 1. Differential Diagnosis of AIGAs Syndrome with Other Diseases

| **Disease Name** | **Usual Age of Onset** | **Pathogenesis** | **Clinical Manifestations** | **Immunological Features** | **Histopathological Features** | **Diagnostic Criteria** | **Management** | **Prognosis** |
| --- | --- | --- | --- | --- | --- | --- | --- | --- |
| **AIGAs Syndrome** | Adult-onset | Autoimmune, anti-IFN-γ antibodies neutralize IFN-γ function, leading to infections | Disseminated mycobacterial/fungal infections, possible VZV/salmonellosis involvement | Elevated anti-IFN-γ autoantibodies | No specific histopathological findings | Serum anti-IFN-γ antibodies detected | Antimicrobial therapy for infections; immunomodulators if necessary | Guarded if infections controlled |
| **MSMD** | Childhood-onset | Genetic mutations in IFN-γ signaling pathway impair immune responses to mycobacteria | Susceptibility to mycobacterial infections (BCG, environmental), possible dissemination | Mutations in genes critical for IFN-γ signaling (e.g., IL12RB1, IFNGR1) | Granulomas in affected tissues | Genetic testing for IFN-γ pathway mutations, clinical/immunological assessments | Immunoglobulin replacement, IFN-γ therapy based on genetic defect | Poor without treatment, due to recurrent infections |
| **GLILD** | Predominantly in adults | A pulmonary complication of CVID, associated with immune abnormalities | Respiratory infections, wheezing, dyspnea, pulmonary function abnormalities | Immune dysregulation with variable antibody deficiencies | Lymphoid interstitial pneumonia (LIP) pattern with granulomas | Clinical symptoms, imaging evidence of pulmonary lymphoid hyperplasia, genetic testing | Antimicrobials for infections; immunomodulators if necessary | Favorable with treatment, though some experience relapses |
| **Sarcoidosis** | Most often affects adults | Granulomatous disease of unknown cause, likely immune-mediated | Lymphadenopathy, pulmonary nodules, cutaneous/skeletal involvement | Altered immune responses with possible Th1/Th2 imbalance | Non-caseating granulomas | Clinical symptoms, imaging (chest X-ray/CT), histopathology | Corticosteroids, immunosuppressants, biologics | Generally favorable with treatment, but some have refractory disease |
| **Lymphoma** | Predominantly in adults | Malignant lymphoid tissue proliferation | Lymphadenopathy, fever, night sweats, weight loss, multiorgan involvement | Clonal lymphocyte proliferation | Lymphoid hyperplasia, atypical lymphocytes, mitotic figures | Lymph node biopsy, imaging, blood tests | Chemotherapy, radiotherapy, immunotherapy | Variable based on histological type and treatment response |
| **IgG4-RD** | Predominantly in adults | Immune-mediated inflammation with IgG4+ plasma cell infiltration | Multiorgan involvement (pancreas, salivary glands, lungs, kidneys) with swelling/pain | Elevated serum IgG4 levels and tissue infiltration of IgG4+ plasma cells. | Fibrosis with IgG4+ plasma cell infiltration and a storiform fibrosis pattern. | Elevated serum IgG4, histopathology showing IgG4+ plasma cells, organ involvement | Corticosteroids for inflammation; immunosuppressants if needed | Generally favorable with treatment, but some have residual dysfunction |

MSMD, Mendelian susceptibility to mycobacterial diseases; BCG, Bacillus Calmette Guerin ; GLILD, Granulomatous Lymphocytic Interstitial Lung Disease ; CVID, Common Variable Immunodeficiency ; IgG4-RD, IgG4-related disease.
